# Interdisciplinary Pediatric Palliative Care Team Involvement in Compassionate Extubation at Home: From Shared Decision-Making to Bereavement

**DOI:** 10.3390/children5030037

**Published:** 2018-03-07

**Authors:** Andrea Postier, Kris Catrine, Stacy Remke

**Affiliations:** 1 Pain Medicine, Palliative Care and Integrative Medicine Program, Children’s Hospitals and Clinics of Minnesota, 2525 Chicago Avenue South, Minneapolis, MN 55404, USA; Kris.catrine@childrensmn.org; 2 Department of Pediatrics, University of Minnesota, Minneapolis, MN 55404, USA; 3 School of Social Work, University of Minnesota, Saint Paul, MN 55404, USA; remke005@umn.edu

**Keywords:** compassionate extubation, psychosocial care, children, palliative care, advance care planning, terminal care, end of life

## Abstract

Little is known about the role of pediatric palliative care (PPC) programs in providing support for home compassionate extubation (HCE) when families choose to spend their child’s end of life at home. Two cases are presented that highlight the ways in which the involvement of PPC teams can help to make the option available, help ensure continuity of family-centered care between hospital and home, and promote the availability of psychosocial support for the child and their entire family, health care team members, and community. Though several challenges to realizing the option of HCE exist, early consultation with a PPC team in the hospital, the development of strategic community partnerships, early referral to home based care resources, and timely discussion of family preferences may help to make this option a realistic one for more families. The cases presented here demonstrate how families’ wishes with respect to how and where their child dies can be offered, even in the face of challenges. By joining together when sustaining life support may not be in the child’s best interest, PPC teams can pull together hospital and community resources to empower families to make decisions about when and where their child dies.

## 1. Introduction

Death inevitably involves uncertainty [1]. When the parents of a child are faced with the devastating prospect of a terminal diagnosis, research has shown that being able to plan and make decisions is most important to them, and engaging in shared end-of-life (EOL) decision-making may lead to better parental adjustment [2,3] after the child’s death [4,5,6]. For many parents, this includes planning the location of their child’s death when possible.

Between 40–60% of pediatric deaths in the intensive care unit (ICU) setting occur following decisions to end life-sustaining therapies [7]. When a child is being maintained by using artificial ventilation in a neonatal or pediatric intensive care unit (NICU/PICU) setting, the option to die at home is usually possible, but all too often, it may not be offered as professionals may not realize this EOL option exists [8,9,10]. A recent systematic review reported that, while some parents may feel more comfortable keeping their child in the hospital or hospice facility, many parents in the USA, Western Europe, and Australia prefer the home setting [11]. Choosing death at home requires careful review of options, anticipatory guidance around expectations, and the availability of capable home-based care resources, all of which pediatric palliative care (PPC) teams are uniquely trained to provide or locate in the community. However, many times opportunities to consult with PPC teams while a child is in the ICU are missed. This may be because health care providers are unaware that home compassionate extubation (HCE) is even an option, due to potential resource-related barriers (e.g., time needed to consult with PPC team, lack of staffing needed to support the HCE, lack of information regarding home based care options and resources), or based upon misconceptions about the role of PPC. However, if consultations with PPC teams are incorporated earlier on in the hospital setting, especially as the goals of care are beginning to shift from a life-sustaining focus to a focus on minimizing suffering, they may be able to help facilitate open communication around EOL planning with families and hospital staff.

Everyone involved in caring for the child should play an active role in the discussion; communicating care and engendering trust that the plans being made reflect continued care and not abandonment. Decision-making requires good communication between the child, family, and all of the professionals that are involved in the child’s life [12]. Whenever possible, parents should be offered all viable options, as well as adequate time to ask questions and make an informed decision. Comprehensive and early communication that involves the opportunity to plan location of death is in line with palliative care principles, and is associated with more favorable outcomes, such as death in the desired location and lower regret about actual location of death [5]. 

Pediatric palliative care teams can offer careful navigation of discussions about the option of HCE with families during a time of distress as they face their child’s EOL. This is a time when families can be assisted to reclaim more family autonomy and intimacy if well planned. Each of the three domains of PPC apply, from early communication and support for complex decision making, to coordination of emotionally and logistically complex care in the home; through bereavement support and helping families and friends make connections to community support services after the child has died. 

In a recent qualitative study of bereaved parents’ experiences with transport home from the ICU for compassionate extubation, parents described it as positive, deeply meaningful, and providing a sense of control and comfort to themselves and other family members [13]. Most articles that address the topic of pediatric home extubation have been written from an intensive care perspective [14,15,16], with emphasis on the development of care pathways [12,15,17,18] and transport logistics [9]. Few peer reviewed articles have been published from a PPC perspective about HCE and the role of PPC teams [19,20]. The critical care-based EOL care guidelines [12,15,17,18] offer suggestions for family-centered planning, pre- and post-transfer, and care during and after the extubation with critical care and community resources. However, these lists of steps do not capture the nuanced aspects of expert communication and coordination that PPC teams are trained to offer around planning, shared decision-making with families and health care teams, the EOL event itself, and the bereavement period. The composure of PPC team members who are familiar with this procedure contributes to an atmosphere of psychological safety and competence instead of crisis. 

There is an opportunity for further collaboration between intensive care teams, hospital-based PPC teams, community-based palliative care, and hospice teams when cure is no longer possible and being home is a priority for the family. An interdisciplinary approach is needed in order to ensure success [2,15,21]. Inter-professional teams are an important component of bridging gaps in care, and PPC teams are uniquely positioned to provide care across environments (e.g., hospital, home) and circumstances (e.g., early decision-making, bereavement support). Pediatric palliative care teams are specifically designed to offer services that ensure family preparation and coping before and after the child’s death. They are comprised of health care staff that are positioned well and are distinctively experienced in offering the three core PPC task-oriented domains as described by Feudtner et al.: problem-solving/decision-making (e.g., working to define goals and hopes of care with families), interventions (e.g., emotional support, anticipatory guidance, and complex symptom management), and logistics (e.g., coordinating care across environments, from hospital to home) [1].

This paper contains two case reports of children of different ages, medical conditions, and family situations that underwent HCE with direct involvement from a PPC program at a large United States (US) tertiary pediatric hospital system (see Table 1 for a summary). Fictional names have been used and some details changed to protect privacy. These case reports highlight the unique spectrum of services PPC teams play throughout the process, from decision-making through bereavement. Feudtner’s [1] core PPC domains serve as a framework for discussion. 

## 2. Case Presentations

### 2.1. Case 1: Teen with Anoxic Brain Injury and Parental Preference for Death Surrounded by Friends and Family at Home

Jane, a 15 year-old female, was transported to the hospital with severe anoxic brain injury following an attempted suicide by hanging. The PPC team was contacted following intubation in the ICU. The PPC team, physician, and social worker met with Jane’s neurologist and ICU attending physician, and arranged a family conference to help the family understand the child’s condition and to discuss goals of care. Options that were discussed over the ensuing days were: organ donation after cardiac death, compassionate extubation in the PICU, tracheostomy/vent placement with observation for any neurologic improvement, or home extubation to allow for natural death. The parents chose home extubation, requesting that the child have time at home with family and friends (which included children of all ages) at the bedside. This required access to an ambulance, a portable vent, suctioning equipment, cleaning supplies, multiple IV medications, and adequate medical and psychosocial support staffing on short notice.

The PPC team arranged for involvement of a home hospice nurse, social worker (MSW), child life specialist (CLS), and respiratory therapist (RT) who was not part of the PPC team. Before the day of the transport, the PPC physician prepared medications for home transport while the CLS and MSW spoke with the family, Jane’s siblings, and friends to help prepare them and answer questions the day before the transport. That evening, the CLS and MSW met to debrief about any concerns they had after meeting with Jane’s family and friends, including an assessment of potential psychosocial challenges that may arise based on their past experiences with HCE. A do not resuscitate (DNR) order was placed in the chart, and all hospice enrollment paperwork was completed. Strategies for managing logistical, financial, and emotional challenges had to be determined quickly (e.g., payment for the ambulance transport and portable vent equipment, which are not covered by insurance).

 *Key Point, Core PPC Domain 1 (Problem-solving and decision-making): This case is an example of how a hospital-based PPC team facilitated medical decision making with the family and medical team & addressed the unique logistical complexities of this case while facing time constraints. The palliative care team gathered an interdisciplinary group of staff members to help facilitate problem-solving and logistical planning by simultaneously drawing upon each team member’s skills.*

The care team met together on the morning that Jane was to go home and included PPC, intensive care, and other hospital staff. They jointly reviewed the planned process in a step-wise fashion, which allowed team members to ask questions and identify additional needs or potential issues. Following this, the team met with family briefly to verify the plan and to inquire about any new psychosocial developments. Jane was transported by ambulance along with the hospice nurse in charge of comfort medications and the RT who was responsible for operating the portable ventilator and suctioning equipment. The PPC physician drove the MSW and a physician fellow with her to Jane’s home separately. A PPC fellow was in attendance in order to learn, and also to field any pages the PPC physician received to ensure dedicated attention to Jane and her family, while simultaneously attending to any urgent calls from other patients and families. 

Jane’s family friend drove her parents to their home from the hospital. The CLS was already at the home with family and friends when Jane arrived to provide support and prepare them for what they were likely to witness. Jane was placed in her own bed with continuous monitoring for comfort and suctioning needs. Once in the home, the MSW and CLS addressed Jane’s loved ones to explain the process in broad strokes and to offer opportunities to say goodbye for those who did not wish to be present during the actual extubation. Those who remained were educated by the physician and nurse about the physical changes that they were likely to witness, including agonal breathing and temperature change. All present gathered at the bedside, a religious blessing was conducted, medications were provided, and the RT removed the ventilator and the endotracheal tube. The PPC team treated excessive secretions with atropine drops and observed for signs of dyspnea or distress (which were not observed) before the PC physician pronounced death 30 min later.

 *Key Point, Core PPC Domain 2 (Interventions): The PPC team, experienced and licensed to practice in the home, was able to bring both medical care and psychosocial support to the child’s family and friends on short notice. The team’s knowledge and experience in providing end-of-life care was critical to being able to orchestrate medical transport, manage symptoms and provide bedside support, thus honoring Jane’s family’s wishes.*

Dark blue blankets, opaque garbage bags, and pressure dressings were used for draping and discretely removing the IV lines and foley catheter so that the secretions and bloodstaining were barely visible. This helped allow family and friends to continue to grieve and console each other without disturbance, and to prevent the potential trauma to the observers. Jane’s mother fled the room at the time that death was pronounced and was consoled by a designated relative and additional friend. The MSW checked in with her after a few minutes and she chose to return to the bedside where Jane’s sister was crying and being supported by her father. Jane’s father addressed everyone that was present, and many of Jane’s high school friends remained in the home consoling each other. During this intensely emotional situation, those who were attending to the immediate family members were able to provide the needed support under the guidance of the psychosocial team members. The total time spent at Jane’s house following the transport was three hours. The MSW, hospice nurse, and CLS stayed until the funeral home came to remove Jane’s body, providing supportive counseling, anticipatory guidance, and acute support. The nurse disposed of the comfort medications, while the PPC physician and RT took the remaining equipment back to the hospital in her car. Bereavement support was arranged including follow-up home visits by the PPC team. The CLS visited Jane’s school to assist with communication and offering grief support resources to classmates.

### 2.2. Case 2: Collaboration with Rural Hospice Agency and Unanticipated Length of Time before Death after Extubation at Home

AJ was an 18 month-old with spinal muscular atrophy (SMA) Type I and respiratory failure. He received a tracheostomy with ventilation at 11 months of age, spending much of his life in the hospital. His family lived in a semi-rural area one hour from the hospital. His family and team recognized he was losing ground to his disease, and his quality of life was deteriorating. They decided that HCE was the best option, but they wanted to be able to spend some time with him at home first. He was referred to the PPC team after this decision had been made at a PICU care conference. After meeting with the family and acknowledging the complex nature of this situation (including the remote geographic location), the PPC team facilitated referrals to a local hospice agency and a home care agency to coordinate support and provide extended hours nursing. The family’s local chaplain was also contacted for additional, familiar support. Both of the agencies had limited to no pediatric experience, including how to provide ventilator support in the home. However, they wanted to help the child and his family, who they considered to be part of their community. 

Prior to the extubation, the hospital-based PPC team played an integral role in the process by providing the local care team with “just in time” training around pediatric hospice and EOL care. The hospice staff had questions about how to care for a child, and the home-based hospice nurses had questions about how to provide EOL care versus the ICU level of home care they were accustomed to. A PPC team MSW and nurse met with local hospice staff on several occasions to provide consultation and support. As the local team became familiar with the situation and the family, their anxieties lessened, and they felt more capable. The PPC team was available by phone as needed, offering 24/7 consultation. The child’s death was planned collaboratively with the family, home nursing agency, and local hospice team. The PPC team sent a hospice nurse, PPC physician, MSW, and chaplain to the family’s home to assist the local teams during the extubation.

 *Key Point, Core PPC Domain 3 (Logistics): This case presented logistical challenges up front due to the family’s rural home location, lack of a local community home hospice team with pediatric experience, PPC staffing limitations due to the distance, and medical transport and supply challenges. The palliative care team served as a bridge between the family and their community resources to equip both with the necessary education and coordination to enable AJ to be at home in a community without pediatric-specific end of life resources. This case presented an opportunity for PPC teaching and network-building in the community.*

It was expected that AJ would die within minutes to hours of extubation, but he lived for two days. This presented some unanticipated challenges to the family and the trusted PPC team staff when it came to leaving the home when the situation had stabilized. When the MSW planned to leave, the parents expressed their distress, so she stayed a couple of hours longer until the family seemed more confident in the support they had; their needs for intimacy with their family taking precedence. After this additional transition time, the family was much more at ease and was comfortable with additional staff taking leave. Home nursing staff remained in the home overnight to provide extra support. Nurses confronted some unanticipated, emergent needs for more morphine, respiratory toilet supplies, and sudden need for flexibility of staff to cover shifts on short notice. Fast planning under pressure was required, away from the family’s view to avoid additional distress for them. While well managed, the unanticipated events resulted in high stress for the team; stress that perhaps would have been lower if some contingency plans had been made. This was an important lesson for the team going forward. AJ ultimately died peacefully with his family surrounding him. The PPC team MSW and nurse held a debriefing meeting with the local care teams. Bereavement support for the family was coordinated through the local hospice agency.

## 3. Discussion

The two case presentations depict how an interdisciplinary PPC team can play an integral role in making HCE possible and successful, even under very different clinical, patient, and family circumstances. Each case report illustrates the importance of flexibility and teamwork across different types of environments and medical and psychosocial providers. Experiences are subjective and nuanced, diverse, and unique to all of the individuals involved, regardless of the person’s role in the process (e.g., staff, family, friends, community members). Success in the context of HCE requires “systems” thinking: noting how the different aspects of the care system interact, where gaps and needs for linkages are surfacing, and how the needs of the child and family can best be addressed. Pediatric palliative care teams routinely focus on complex communication, decision making, creative problem solving, and advocacy for the preferences of patients and their families. We recommend that intensive care staff consider consulting with PPC teams as soon as continued ventilator support is deemed non-beneficial by the family and care team. See Table 2 for a list of key considerations when considering HCE.

The essential steps in the process of HCE presented in the two cases above can be described using Feudtner’s [1] three core palliative care domains framework, with particular attention to the unique psychosocial support PPC teams can offer in the home to patients, their families, and health care teams during a time that the child’s family will remember for the rest of their lives. 

### 3.1. Problem-Solving/Decision-Making

Pediatric palliative care teams are trained to offer family-centered care and comprehensive communication throughout the decision-making and bereavement process, and across environments of care [1,22]. Communication involves depiction and detection; reframing and re-anchoring. In the context of EOL decisions, each of these dimensions occurs in a situation fraught with complexity, emotion, and high stakes for the family, as well as the whole team of health care providers, including hospital and home based practitioners. For example, when AJ lived longer than anticipated, it was important to reassess and adjust the plan on short notice in order to ensure continued safe care in the face of quickly diminishing resources. Focus was on accessing needed people, plans, and supplies in the background so that the situation did not evolve into an unnecessary crisis for the family. Communication with the family focused on reassurance and offering updates in a reassuring manner. Communication was specific and direct, reducing the potential for additional family distress.

The majority of families prefer a shared decision-making model when faced with critical choices. This has been well established in previous research [23,24,25,26]. However, there remain some families who prefer to make decisions autonomously or to defer entirely to their medical teams. The gravity of these choices warrant the expertise of a PPC team trained to assess the specific family’s preferences and facilitate decision-making discussions. The importance of this involvement is elevated in situations for which there are additional complicating factors, such as family discord, reluctance to engage in decision-making, lack of alignment between family needs and the child’s care team’s (e.g., ICU staff) comfort in discussing the issue, and the lack of sufficient time to engage in lengthy discussions. One of the most important aspects of care at this EOL phase is ensuring that the best possible decision-making process occurs in order to minimize the potential for regrets later, and to facilitate future clarification in the event that new questions or concerns arise.

Shared decision-making involves more than simply shared understanding of facts and exchanges of information. It is a process that encompasses emotional, relational, and psychological intensity with “high stakes” implications for the family and their integrity and identity as a family unit going forward [1,27]. All of this unfolds within a context of uncertainty, grief, and the compelling nature of unique situations. Early referral to PPC may enable this process to unfold over time, as complex decisions often require “sitting with facts” and processing emotions as clarity emerges. Trust between the team and the family evolves through this process, underscoring the value of time and patience. Collaboration between the attending team, the PPC team and the family can promote opportunity for successful transfer of the family’s trust to future health care teams. Therapeutic relationships with skilled clinicians who possess a thorough understanding of the situation can offer the family a “container” in one sense, and can contribute to establishing psychological safety for the family at a critical time when there is high potential for trauma and unintended, negative outcomes. Skilled clinicians, such as MSWs, must be prepared to anticipate potential events in order to manage them successfully. In the context of HCE, this means minimizing family anxiety and their sense of uncertainty, and also reinforcing a sense of control in the midst of a situation that feels inherently out of control.

Real, active involvement of parents in conversations is critical, as is family-centered care with adequate staffing to accommodate it. For example, Jane’s family chose to include family and friends at the bedside at the time of the extubation, creating the notable challenges of helping to support many people who are at varying developmental stages, enlisting family and community members to support each other during their own grief experience, and preparing for the potential of emotional crisis in several attendees simultaneously. The CLS and MSW were scheduled on short notice to provide counseling support for anticipatory grief, decision-making, and to offer support to the family and friends of the dying child. This support extended beyond the extubation to being present and ready to manage unanticipated events, while remaining off to the side so that friends and family could console each other. The presence of the PPC team members can also offer families and staff a sense of reassurance that events are unfolding as they should. In Jane’s case, the CLS and MSW provided coaching to the health care team and family about how best to respond to the patient, and empowered the family to proactively ask the health care team to address any needs they felt the child had in her last moments of life. This provided an opportunity for family to honor their child’s life by gathering with loved ones in a setting and manner consistent with how her previous milestones in life had been honored, and to create a shared memory of the event as a lasting legacy. The family viewed Jane’s death as dignified, in part due to the active prevention of suffering and presence of the PPC team in Jane’s home setting alongside her community of friends and family. 

### 3.2. Interventions

Depth and breadth of psychosocial services that are available to the patient, parents, siblings, and the family’s community (e.g., school) ensures holistic, appropriate care across settings and time. The range of services that families and their children often need include anticipatory guidance, supportive counseling, episodic problem-solving assistance, parent guidance, age appropriate information and education for siblings, friends and neighbors, reinforcement of coping, and crisis management assistance. As indicated in both case examples, unanticipated events and inexperienced people that are involved in providing EOL care required the presence and active guidance by experienced PPC staff. The MSW is charged with attending to the family’s well-being, providing supportive counseling, carrying out assessments of emotional and psychological issues that require intervention, and maintaining a systems-oriented perspective on practical management of the entire situation. The CLS provides anticipatory guidance, age appropriate information and preparation, age appropriate activities for children present, legacy-oriented engagement, and emotional support to the patient and any children present. A chaplain can provide religious and/or spiritual support and reassurance at a critical time, often collaborating with community clergy who might not have experienced a child’s death before.

In addition to psychosocial support for HCE, medical expertise can be offered by PPC physicians and nurse practitioners (NP), as well as hospice nursing staff. Pediatric palliative care team physicians and NPs have experience anticipating symptoms, titrating comfort medications in proportion to a child’s changing symptom burden while reassuring families that symptoms are being continuously managed, can prepare families for likely physical changes around the time of death, and can help dispel myths about hastening death or other ethical concerns that families may have. Experienced PPC nurses can ensure adequate supply and equipment management, anticipate potential issues with the technical aspects of the HCE plan, provide expert symptom assessment, help to minimize the visual impact of needed cares on the family, and help to establish a close and trusting working relationship with the family.

Diverse family situations require sensitive and individualized responses from the care team. The only way to achieve this is through engaging a process that emphasizes nuanced communication and confident clinical practice around EOL care. Attending to a child at the EOL requires a hybrid approach that blends clinical expertise with counseling, education, and support that travels in many directions at once. For example, in AJ’s case, care went into providing support not only to the patient and family, but also to staff from the two agencies that were brought in to provide care in the home. Consideration with respect for the feelings of the PICU team that discharged AJ was important, acknowledging the fact that they were very emotionally invested in him and his family through the care they had provided. Their trust in the PPC team in turn inspired the family’s ability to trust the PPC team. The PPC team was also aware of the significance that AJ’s broader home community network would play in supporting the family in their bereavement over time. Everyone invested in caring for AJ and his family along the way would benefit from psychosocial support. 

There is sometimes concern that family may panic and decide not to allow the discontinuation of the ventilator once at home. This requires a MSW readiness assessment in advance, but unexpected reactions may still emerge in the moment. In Jane’s case, the large crowd of friends and family of different ages was a factor for the medical team to plan for. What if something went wrong? What if distressing symptoms emerged? Planning for such unanticipated events includes social work, child life, chaplaincy and others (e.g., music therapy, volunteers) as best practice. This support extends beyond the child’s immediate family (friends, school, other health care workers) to include their circle of support in the community.

### 3.3. Logistical Efforts

Collaborating across sites and units for care involves complex steps and processes. We are not just bridging a gap and promoting continuity of EOL care, but when PPC already has an established relationship with the NICU, PICU, and other services, practitioners can link across relationships to promote trust and logistical safety. Finesse involved in managing from behind the scenes without family/friends being aware—bodily fluids; crisis of unexpected time to die and morphine shortage—while managing the complex array of emotions and relationship challenges is required. Active problem solving and intervention around emerging issues is required on the fly. Experience and expertise is essential. Sometimes children live longer than expected after the ventilator is removed [28], like AJ. Parallel planning should take place, as a recent study found one third of children transferred from an intensive care setting to a hospice for EOL survived beyond two weeks [20] and a recent literature review of six case series and reports on pediatric critical care transports from ICU to home for terminal extubation showed that, while the vast majority of infants and children died within hours to days after extubation, one study reported two children living as long as 17 and 40 days [9].

If hospital staff have good experiences collaborating with PPC to help grant parents their wishes, staff will be more likely to engage PPC in conversations in the intensive care setting and feel good about being able to offer a choice—a “good death”—to parents in the future. For example, several months after AJ’s death, another pediatric case was referred to the same local hospice. They readily accepted the referral because they felt so gratified by their PPC program. Support meetings were also offered, but the local team felt able to handle the situation with phone consultations as needed, thanks to the relationship formed between the PPC staff and local hospice team during AJ’s case. 

### 3.4. Staffing/Resources

Compassionate extubation at home requires both physical and staffing resources. Palliative care teams may be helpful in planning for the support systems required. Jane’s care brought several resource-related issues to light that can serve to inform the practice of HCE going forward. First, her size and condition required ambulance transportation rather than by private family vehicle. This is not normally an insurance-covered expense and can be cost-prohibitive for many families. Our MSW team has successfully sought assistance through “wish” and charity organizations for some families, but this assistance may not be equally available to all of the children because of their diagnosis. Additionally, some families request the opportunity to spend a prolonged period of time at home before extubation, which requires having a transport ventilator in the home. 

Staffing coverage can be an issue, especially on weekends. In Jane’s case, the physician was on call the day of the extubation, so she brought a PPC physician fellow along to answer any pages during the event. The logistical issues of being exclusively present for a child and family around the time of extubation can be challenging to navigate when a single physician is on call for many other patients. In Jane’s case, the PPC fellow was able to assist with other patient needs, but many PPC services do not have trainees or the availability of backup staffing. Finally, the availability of MSW and CLS support was critical in Jane’s case, as a large number of teenage friends were present. Availability of these services can be limited depending upon the day of the week, or dependent on the availability of such resources in a community without a dedicated home-based PPC and hospice program.

It is important to note that the children described in this study were cared for in the US context, where a lack of insurance and financial stability exist, resulting in inability to support a hospice home model [34] that is more commonly available in the UK, Europe, and Canada. Lack of a hospice home option may reduce the likelihood in the US of viewing HCE as a viable option. 

## 4. Summary

With careful planning and collaborative goal-setting, parents can usually be given a choice about where their child dies. There are often great collaborative opportunities for intensive care and PPC teams to make this happen. Ideally, intensivists would contact the PPC team before continued/active treatment has been deemed unhelpful and distressing to the child and family. Early engagement of PPC encourages positive and trusting relationship-building, opportunities for presenting options to the family, and giving families the space they need to actively participate in EOL decision-making for their child. Pediatric palliative care teams are uniquely positioned to bridge resources between the hospital and community as a result of specialized training in dealing with psychosocial issues during the EOL and bereavement periods. We hope that this article encourages timely collaboration between intensive care teams and PPC teams, with the shared goal of involving parents at every stage of the decision-making process in order to successfully grant the wishes of parents in a tragic situation.

## Figures and Tables

**Table 1:** Patient and Family Characteristics.

Case	Age	Sex	Medical History	Days on Ventilator	PPC Team Involvement	Non-PPC Staff Involvement	Time to Death Following Extubation	Challenges
1	15 years	F	Severe anoxic brain injury secondary to attempted suicide by hanging	2 days	Physician, nurse, MSW, CLS	RT, chaplain	30 min	Portable vent; ambulance transport cost; physician on-call demands; CLS challenges; large and varied family/community “audience”
2	18 months	M	Spinal muscular atrophy, Type I	8 months	Physician, MSW, nurse, chaplain	Community hospice nurses (adult-focused), pediatric home care nurses, local chaplain	7 days	Inexperienced local hospice; training needs; psychosocial support needs; collaboration with two remote agencies; unanticipated (longer) time to death

**Table 2:** Key Considerations Prior to Home Compassionate Extubation in Infants and Children with the Possibility of Continued Life at Home.

Topic	Things to Consider
1. Medication supply	✓ Who will provide initial supply and refill or prescribe new medications if needed (e.g., child lives longer than expected)? Where will they be filled? How will they be discarded after death?
2. Transportation	✓ Can the child be taken in private vehicle, or will she require medical transportation? Who will drive? Are there arrangements to transport needed staff and family?
3. Medical equipment	✓ Will suction or a portable vent be needed? How will equipment be returned to the hospital after death?
4. Staffing in the child’s home	✓ Anticipate all possible psychosocial support staffing needs in the home before/after HCE (e.g., CLS, MSW, chaplain). ✓ How long will staff (e.g., nurses) be able to remain in the home? If needed, is there a plan for staff relief? ✓ If collaborating with a community home hospice team, is training necessary (e.g., rural hospice team with no pediatric experience)? ✓ Consider communication and collaboration with child’s community-based pediatrician as appropriate.
5. Financial and legal	✓ HCE may pose additional legal consequences for some stakeholders and as such should be carefully considered. However, this should not be the overriding concern when the focus is on what is best for the child. Consider an ethics consultation if necessary. ✓ Ensure insurance or private payment is available to cover the cost of ambulance transport, ventilator, home nursing, IV medications, etc.). Check for gaps in insurance coverage if transitioning to a community-based resource. ✓ If child has life insurance plan, death must be designated as due to a disease process rather than as assistance in dying for benefits to be paid. ✓ Provide documentation of anticipated death in a format that meets local legal and community standards for advanced directives. These could include MD letter, POLST or other, and vary by jurisdiction. ✓ If child is in state custody or foster care, check to see if specific regulations apply.
6. Autopsy and organ donation	✓ Ensure family is well informed about organ and tissue donation options and limitations (e.g., cost of autopsy and additional transportation).
7. Anticipatory guidance for the family	✓ Prepare family for what they will witness at end of life (i.e., sights, sounds, smells) [28]. Provide information at the appropriate developmental level and paced to match the emotional needs of family members and friends. ✓ Discuss feeding and hydration with child’s family in advance. Consider discontinuation of artificial nutrition and hydration in patients who are unconscious, or if likely to cause burdensome symptoms [29]. Consider feeding/hydration to comfort for those who are wakeful and express hunger or thirst. ✓ Consider referring parents/staff to additional support/educational resources. Examples are videos Choosing Thomas—Inside a family’s decision to let their son live, if only for a brief time [30] and Making Every Moment Count [31]; the online support site Courageous Parents Network [32], and Cameron’s Arc: Creating a Full Life: Teaching and Resource Guide [33].
8. Prognostication	✓ Discuss the likely length of life after extubation, but also prepare for living longer than expected. Confirm family’s wishes to continue care at home versus re-hospitalization or transport to a hospice facility.
9. Plan priorities for the child’s time at home	✓ Discuss how family wishes to spend their time at home with the child, incorporating any rituals or important practices, and adjusting the medical plan as needed to accommodate. Plan and allow for legacy activities, such as photos, videos, hand/foot prints, locks of hair, as well as storytelling, reminiscing, etc.
10. Comprehensive assessment of the family’s emotional, psychological and practical capacity for managing the compassionate extubation event in their home.	✓ In most instances emergency mental health resources will be unfamiliar with this unique sequence of events. Therefore, the PPC team should be prepared to manage unanticipated emotional or psychological crises that may occur. However, in practice we have not observed this to be a significant concern with careful planning.

## References

[1] Feudtner C. (2007). Collaborative communication in pediatric palliative care: A foundation for problem-solving and decision-making. Pediatr. Clin. N. Am..

[2] Longden J.V., Mayer A.P. (2007). Family involvement in end-of-life care in a paediatric intensive care unit. Nurs. Crit. Care.

[3] Lauer M.E., Mulhern R.K., Schell M.J., Camitta B.M. (1989). Long-term follow-up of parental adjustment following a child’s death at home or hospital. Cancer.

[4] Siden H., Miller M., Straatman L., Omesi L., Tucker T., Collins J.J. (2008). A report on location of death in paediatric palliative care between home, hospice and hospital. Palliat. Med..

[5] Dussel V., Kreicbergs U., Hilden J.M., Watterson J., Moore C., Turner B.G., Weeks J.C., Wolfe J. (2009). Looking beyond where children die: Determinants and effects of planning a child’s location of death. J. Pain Symptom Manage..

[6] Friedrichsdorf S.J., Postier A., Dreyfus J., Osenga K., Sencer S., Wolfe J. (2015). Improved quality of life at end of life related to home-based palliative care in children with cancer. J. Palliat. Med..

[7] Burns J.P., Rushton C.H. (2004). End-of-life care in the pediatric intensive care unit: Research review and recommendations. Crit. Care Clin..

[8] Craig F., Mancini A. (2013). Can we truly offer a choice of place of death in neonatal palliative care?. Semin. Fetal Neonatal Med..

[9] Noje C., Bernier M.L., Costabile P.M., Klein B.L., Kudchadkar S.R. (2017). Pediatric Critical Care Transport as a Conduit to Terminal Extubation at Home: A Case Series. Pediatr. Crit. Care Med..

[10] Meert K.L., Sarnaik A.P. (2010). Choosing between death at home or in the hospital: Respecting the principle of autonomy. Pediatr. Crit. Care Med..

[11] Bluebond-Langner M., Beecham E., Candy B., Langner R., Jones L. (2013). Preferred place of death for children and young people with life-limiting and life-threatening conditions: A systematic review of the literature and recommendations for future inquiry and policy. Palliat. Med..

[12] Sine D., Sumner L., Gracy D., von Gunten C.F. (2001). Pediatric extubation: “Pulling the tube”. J. Palliat. Med..

[13] Nelson H., Mott S., Kleinman M.E., Goldstein R.D. (2015). Parents’ Experiences of Pediatric Palliative Transports: A Qualitative Case Series. J. Pain Symptom Manag..

[14] Simpson E.C., Penrose C.V. (2011). Compassionate extubation in children at hospice and home. Int. J. Palliat. Nurs..

[15] Laddie J., Craig F., Brierley J., Kelly P., Bluebond-Langner M. (2014). Withdrawal of ventilatory support outside the intensive care unit: Guidance for practice. Arch. Dis. Child..

[16] Needle J.S. (2010). Home extubation by a pediatric critical care team: Providing a compassionate death outside the pediatric intensive care unit. Pediatr. Crit. Care Med..

[17] Oppenheim S., Bos C., Heim P., Menkin E., Porter D. (2010). Developing guidelines for life-support therapy withdrawal in the home. J. Palliat. Med..

[18] Cottrell S., Edwards F., Harrop E., Lapwood S., McNamara-Goodger K., Thompson A. (2011). A Care Pathway to Support Extubation within a Children’s Palliative Care Framework.

[19] Zwerdling T., Hamann K.C., Kon A.A. (2006). Home pediatric compassionate extubation: Bridging intensive and palliative care. Am. J. Hosp. Palliat. Care.

[20] Gupta N., Harrop E., Lapwood S., Shefler A. (2013). Journey from pediatric intensive care to palliative care. J. Palliat. Med..

[21] Hawdon J.M., Williams S., Weindling A.M. (1994). Withdrawal of neonatal intensive care in the home. Arch. Dis. Child..

[22] Jones B.L., Contro N., Koch K.D. (2014). The duty of the physician to care for the family in pediatric palliative care: Context, communication, and caring. Pediatrics.

[23] Meert K.L., Thurston C.S., Sarnaik A.P. (2000). End-of-life decision-making and satisfaction with care: Parental perspectives. Pediatr. Crit. Care Med..

[24] Kon A.A. (2011). Life and death choices in neonatal care: Applying shared decision-making focused on parental values. Am. J. Bioeth..

[25] Sharman M., Meert K.L., Sarnaik A.P. (2005). What influences parents’ decisions to limit or withdraw life support?. Pediatr. Crit. Care Med..

[26] Lipstein E.A., Brinkman W.B., Britto M.T. (2012). What Is Known about Parents’ Treatment Decisions? A Narrative Review of Pediatric Decision Making. Med. Decis. Mak..

[27] Browning D.M., Solomon M.Z. (2006). Relational learning in pediatric palliative care: Transformative education and the culture of medicine. Child Adolesc. Psychiatr. Clin. N. Am..

[28] Catlin A., Carter B. (2002). Creation of a neonatal end-of-life palliative care protocol. J. Perinatol..

[29] Hellmann J., Williams C., Ives-Baine L., Shah P.S. (2013). Withdrawal of artificial nutrition and hydration in the neonatal intensive care unit: Parental perspectives. Arch. Dis. Child. Fetal Neonatal Ed..

[30] The Dallas Morning News Choosing Thomas—Inside a Family’s Decision to Let Their Son Live, If Only for a Brief Time. https://www.youtube.com/watch?v=ToNWquoXqJI.

[31] National Film Board of Canada Making Every Moment Count. http://www.nfb.ca/film/making_every_moment_count/.

[32] Courageous Parents Network. https://courageousparentsnetwork.org/.

[33] American Academy of Pediatrics Cameron’s Arc: Creating a Full Life; Teaching and Resource Guide. https://shop.aap.org/camerons-arc-creating-a-full-life/.

[34] National Hospice and Palliative Care Organization (NHPCO) (2015). NHPCO’s Facts and Figures: Pediatric Palliative and Hospice Care in America.

